# Case Report: Successful treatment of steroid-refractory immune checkpoint inhibitor associated myocarditis with tofacitinib

**DOI:** 10.3389/fimmu.2026.1778284

**Published:** 2026-03-31

**Authors:** Defei Kong, Feng Zhang, Yanxia Liu, Xiaojuan Zhu, Ting Yuan, Anli Liu, Lulin Zhou, Wen Chen, Qingmin Yao

**Affiliations:** Department of Geriatrics, Shandong Provincial Hospital Affiliated to Shandong First Medical University, Jinan, China

**Keywords:** immune checkpoint inhibitors, immune-related adverse events, Janus kinase inhibitors, multiple organ injury, steroid-refractory immune checkpoint inhibitor associated myocarditis, tofacitinib

## Abstract

The advent of immune checkpoint inhibitors (ICIs) has revolutionized cancer treatment but is associated with immune-related adverse events (irAEs) that can involve multiple organ systems.Steroid-refractory immune checkpoint inhibitor associated myocarditis, defined as persistent or progressive myocardial injury despite high-dose corticosteroids requiring escalation of immunosuppression, represents a life-threatening clinical challenge with limited therapeutic options. This case report describes a 75-year-old female patient with deficient mismatch repair sigmoid colon adenocarcinoma who developed severe multi-organ irAEs, including myositis, hepatitis, and steroid-refractory myocarditis 25 days after receiving QL1706 (a PD-1/CTLA-4 bispecific antibody). Initial intervention with high-dose glucocorticoid pulse therapy combined with intravenous immunoglobulin failed to control the myocardial injury. The clinical course was further complicated by endocrine involvement and hematological toxicity following second-line immunosuppression with mycophenolate mofetil. After tofacitinib administration, the patient’s clinical symptoms and laboratory findings showed significant improvement. Notable adverse effects included oral mucositis, diarrhea, and a pulmonary fungal infection, which were managed supportively. This case highlights the potential role of tofacitinib in managing complex, steroid-refractory multi-organ irAEs. However, the significant infection risk underlines the necessity for vigilant monitoring and prophylactic strategies. Further prospective studies are needed to define the efficacy and safety of JAK inhibitors in this setting.

## Introduction

The advent of immune checkpoint inhibitors (ICIs) has ushered in a new era in tumor immunotherapy, significantly improving response and survival rates across multiple malignancies. ICIs mainly block the interaction between immune checkpoint proteins, relieve the inhibition of the immune system by tumor cells, and reactivate the ability of immune cells to kill tumor cells. Although immune checkpoint blockade can effectively combat tumors by enhancing immune system activity, it may also induce inflammatory side effects, commonly referred to as immune-related adverse events (irAEs) (1). IrAEs can involve almost all organ systems, with the most commonly affected sites being the gastrointestinal tract, endocrine glands, skin, and liver. Other reports have indicated that the central nervous system, cardiovascular system, lungs, musculoskeletal system, and hematological system may be involved (2). Although the incidence is low, the associated risks are high. Here, we report a case of steroid-refractory immune checkpoint inhibitor associated myocarditis, complicated by myositis and hepatitis, in a patient with deficient mismatch repair (dMMR) sigmoid colon adenocarcinoma following treatment with QL1706. This report focuses on steroid-refractory myocarditis and highlights the potential role of tofacitinib in such cases, aiming to provide a reference for clinical management.

## Case presentation

A 75-year-old female presented in June 2025 with left lower abdominal pain and a sensation of incomplete defecation for more than 20 days. She had a history of hypertension and diabetes. Colonoscopy revealed a circumferential nodular lesion in the sigmoid colon (20–23 cm from the anal verge), and pathology confirmed sigmoid colon adenocarcinoma with deficient mismatch repair (dMMR) and PD-L1 (22C3) combined positive score (CPS) = 10. Pretreatment staging assessments including colonoscopy, contrast-enhanced abdominal/pelvic computed tomography (CT) and chest CT confirmed the clinical stage as cT3N2M0, with no evidence of distant metastasis. After multidisciplinary discussion and informed consent, she was enrolled in a clinical trial and received intravenous infusion of QL1706 300mg on June 27, 2025(defined as day 0 for the following clinical course description).

Approximately 25 days after treatment (July 22, 2025), she was readmitted to our hospital due to persistent systemic myalgia, fatigue, chest tightness, and eyelid edema. Laboratory tests revealed severe liver injury (aspartate aminotransferase(AST) 282 U/L, normal 13–35 U/L; alanine aminotransferase(ALT) 183 U/L, normal 7–40 U/L) and significant myocardial injury(creatine kinase (CK) 6523 U/L, normal 40–200 U/L; creatine kinase-MB (CK-MB) 223.70 ng/ml, normal 0.6-6.3 ng/ml; myoglobin (MYO) 3297.30 ng/ml, normal 14.3-65.8 ng/ml; high-sensitivity troponin T (hs-TnT) 779 pg/ml, normal 0-17.5pg/ml). The electrocardiography (ECG) showed ST-segment depression in leads V2 and V3, and T-wave flattening in V3–V5, while the baseline ECG performed prior to QL1706 infusion on June 25, 2025, showed a normal sinus rhythm without ST–T segment abnormalities or pathological Q waves. Cardiac Doppler ultrasound indicated segmental wall motion abnormality, aortic sclerosis with mild regurgitation, and left ventricular ejection fraction (LVEF) of 60%. Cardiac magnetic resonance imaging, coronary angiography, and endomyocardial biopsy were not performed because the patient was critically ill and unable to tolerate invasive procedures. Myocardial injury developed 25 days after immune checkpoint inhibitor therapy, establishing a clear temporal association. Markedly elevated cardiac biomarkers, together with new electrocardiographic and echocardiographic abnormalities, supported the clinical diagnosis of ICI associated myocarditis, and ischemic myocardial injury was considered unlikely. Due to the severity of her condition, she was immediately admitted to the intensive care unit. Multidisciplinary consultation confirmed immune-related adverse events, including immune-mediated myocarditis, myositis and hepatitis.

On day 26(July 23, 2025), pulse methylprednisolone (500 mg/day for 3 days) was initiated along with supportive hepatoprotective therapy, including magnesium isoglycyrrhizinate, glutathione, and polyene phosphatidylcholine, which were used to stabilize hepatocyte membranes, reduce oxidative injury, and promote hepatic recovery. Her myalgia improved rapidly, with marked declines in CK-MB, MYO, and liver enzymes (e.g. CK-MB decreased to 97.7 ng/ml, MYO to 733 ng/ml, hs-TnT to 387 pg/ml). Methylprednisolone was then tapered to 250 mg/day, and intravenous immunoglobulin (IVIg) 20 g/day was administered for 7 days, together with voriconazole for antifungal prophylaxis. However, cardiac biomarkers (hs-TnT, MYO) showed a plateau after initial partial reduction (hs-TnT around 350 pg/ml, MYO around 300 ng/ml) without further decline. By day 36, myalgia had resolved, but she still experienced fatigue and episodic chest tightness, accompanied by hyponatremia (serum sodium 129.1 mmol/L, normal 135–145 mmol/L).The presence of hyponatremia and a decreased serum cortisol level (33.90 nmol/L, normal 166–507 nmol/L) in the context of prolonged high-dose corticosteroid therapy raised concern for secondary adrenal insufficiency.

Given the limited response to steroids and IVIg, methylprednisolone was reduced to 180 mg/day (with a planned taper) and mycophenolate mofetil (MMF) 0.5 g twice daily was added as second-line immunosuppression. Over the following three weeks, CK normalized and skeletal muscle injury resolved, but hs-TnT remained elevated at around 280 pg/ml, indicating persistent myocardial injury. On day 57, hs-TnT rebounded to 424 pg/ml. Concurrently, she developed hypothyroidism (Free Triiodothyronine (FT3) 1.54 pmol/L, normal 3.1-6.8 pmol/L; Free Thyroxine (FT4) 11 pmol/L, normal 11.9-21.6 pmol/L; Thyroid-stimulating hormone (TSH) 2.41 mIU/L, normal 0.27-4.20 mIU/L), suggesting immune-related endocrine dysfunction (possibly hypophysitis or thyroiditis), and levothyroxine replacement was initiated. By day 63, pancytopenia emerged (white blood cell (WBC) count 1.62–2.09×10^9^/L, normal 3.5-9.5×10^9^/L; hemoglobin (HGB) 95–98 g/L, normal 115–150 g/L; platelet (PLT) count 31–65×10^9^/L, normal 125-350 ×10^9^/L); bone marrow examination indicated hyperplasia with anemia and thrombocytopenia, possibly related to MMF or immune-mediated myelosuppression.

**Figure 1:**
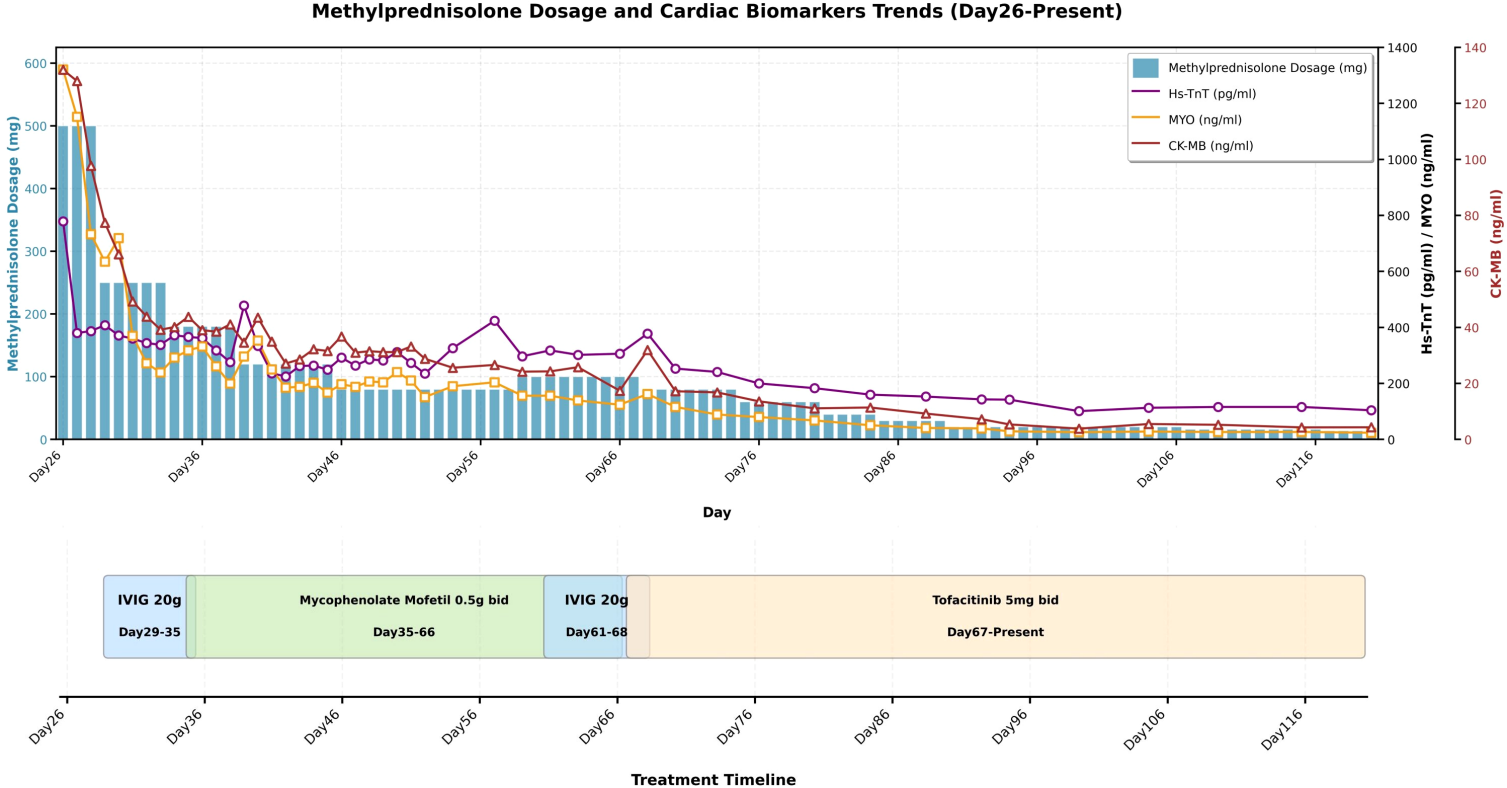
Variation in cardiac biomarkers and treatment strategy with ICI associated myocarditis.QL1706 infusion on 2025.06.27 was defined as Day 0; methylprednisolone pulse administration on 2025.07.23 was Day 26.Hs-TnT, high-sensitivity cardiac troponin (pg/ml);MYO, myoglobin (ng/ml); CK-MB, creatine kinase-MB (ng/ml); IVIG, intravenous immunoglobulin.

Due to inadequate response to high-dose steroids and MMF with ongoing myocardial injury, a diagnosis of steroid-refractory immune checkpoint inhibitor associated myocarditis was established. On day 67(September 2, 2025), MMF was discontinued, and the Janus kinase (JAK) inhibitor tofacitinib 5 mg twice daily was added to methylprednisolone (80 mg/day). Following tofacitinib initiation, cardiac biomarkers showed a clear and significant downward trend. After 32 days of treatment, hs-TnT decreased from 387 pg/ml to 101 pg/ml, and CK-MB declined from 31.9 ng/ml to 3.85 ng/ml. Variation in cardiac biomarkers and treatment strategies are demonstrated in **Figure 1**. Serial ECGs obtained with close monitoring during tofacitinib therapy also showed stepwise improvement. Before tofacitinib initiation, the ECG demonstrated mild ST-segment depression in leads V4–V6 with flattened T waves. On 11 days of therapy, the ST-segment depression had partially resolved, with mild T-wave inversion in leads V2–V3.By 39 days, the ST–T abnormalities had completely resolved, with stable sinus rhythm (65 bpm) and no new ECG abnormalities.

During this period, the patient developed pharyngalgia, severe oral ulceration or mucositis (2–3 days after starting tofacitinib), and worsened diarrhea (1 week later), likely related to tofacitinib, which improved with supportive care. Due to significant elevations in serum fungal G-test and GM-test, voriconazole was switched to intravenous administration. Around 4 weeks into tofacitinib therapy, she experienced infectious fever, which was controlled with appropriate antimicrobial treatment. No further liver enzyme elevation occurred throughout the tofacitinib treatment period. The patient remains hospitalized for ongoing management. Antitumor therapy has been temporarily suspended due to severe multi-organ injury. As of December 25, 2025 (Day 181), abdominal computed tomography revealed no evidence of tumor progression.

## Discussion

ICIs block the binding of the immune checkpoint receptor PD-1 to its ligand PD-L1, or the interaction between CTLA-4 and B7 molecules, thereby relieving the body’s immune suppression, restoring and enhancing the activation and effector functions of tumor-specific T cells, and ultimately promoting the immune system’s recognition and elimination of tumor cells (3). With the expanding clinical use of ICIs, significant survival benefits have been achieved in many malignancies, however, the incidence of irAEs has also increased, which can involve multiple organ systems.

Although the overall incidence of ICI associated myocarditis is low (approximately 0.06%-1.14%), the mortality rate can be as high as 39.7%-66.0% (4). The clinical manifestations are often non-specific, making early monitoring of myocardial injury markers (such as troponin and creatine kinase-MB) and ECG crucial for timely diagnosis and differential assessment (5). In this patient, the clinical diagnosis of ICI associated myocarditis was based on noninvasive findings, a clear temporal association with ICIs therapy, and exclusion of ischemic heart disease, representing a pragmatic and safe approach in critically ill patients unable to undergo invasive evaluation. The incidence of myositis in patients receiving ICIs treatment is approximately 0.6%, and it is often concurrent with other muscle-related irAEs. About 16%-40% of these patients also have myocarditis or myasthenia gravis. Muscle pain or weakness accompanied by elevated myoglobin can assist in diagnosis (6). ICI-related liver injury is mostly asymptomatic, and typically presents with elevated aspartate aminotransferase(AST) and alanine aminotransferase(ALT) levels (1). Most patients can achieve remission after receiving hepatoprotective and immunosuppressive therapy. Therefore, for patients with multiple organ involvement, treatment strategies needs to balance immunosuppression and disease control among various organs.

In the present case, a patient with dMMR sigmoid colon cancer developed multiple organ injury, including steroid-refractory myocarditis 25 days after receiving QL1706 (a bispecific antibody targeting both PD-1 and CTLA-4).Compared with single-target immune checkpoint inhibitors, dual-target therapy may carry a higher risk of multi-organ irAEs (7). And dMMR tumors typically carry a high tumor mutational burden, a biological feature that has been associated with an increased risk of irAEs (8), although the underlying mechanisms remain incompletely understood and warrant further investigation.

Current research suggests that the pathogenesis of ICI associated myocarditis may be related to the presence of common high-frequency T cell receptor sequences in myocardial and tumor tissues. After ICI treatment, activated T lymphocytes targeting tumors may also cross-recognize shared antigens in skeletal muscle and myocardium. The misdirected response can lead to tumor-specific T cells attacking cardiomyocytes (5, 9). This mechanism could explain the co-occurrence of myocarditis and myositis in this patient. Concurrently, immune cells(particularly T cells) activated by ICIs release a large number of pro-inflammatory factors, which directly or indirectly cause cardiomyocyte damage (10). Glucocorticoids are recommended as the first-line therapy for severe irAEs in multiple clinical guidelines (11–13). However, glucocorticoids non-specifically inhibit the inflammatory response, which cannot precisely block the activation and proliferation of certain autoreactive T cells or reduce the production of autoantibodies. Therefore, they are ineffective against some steroid-refractory myocarditis.

For patients with poor response to glucocorticoid therapy, guidelines recommend immunopotentiative therapy, such as infliximab, mycophenolate mofetil, antithymocyte globulin, or tacrolimus (12, 13). In addition, immunotherapeutic drugs including abatacept, alemtuzumab, and tocilizumab have shown potential efficacy in published case reports (14–16). Mycophenolate mofetil (MMF) was initially administered as the second-line immunosuppressive therapy for the patient in this case. However, MMF primarily inhibits B and T cell proliferation and has a limited effect on suppressing the immune response in myocardial tissue (17). Additionally, the development of myelosuppression prevented its continued use.

Recently, Janus kinase(JAK) inhibitors have garnered increasing attention for the management of irAEs (18, 19). JAK proteins are key signal transduction molecules downstream of various cytokine receptors. After cytokines (such as interleukins, interferons, and GM-CSF) bind to their receptors, JAK tyrosine phosphorylation and activation are triggered, which in turn activates signal transducers and activators of transcription(STAT) (20). JAK inhibitors can competitively block the ATP-binding site of JAK, inhibiting its kinase activity and thereby interrupting cytokine-mediated JAK-STAT signaling. Even in the presence of pro-inflammatory factors or immune activators, downstream signal transmission cannot be effectively completed, thereby significantly reducing inflammation driven by various pro-inflammatory cytokines and immune cytokines. In addition, JAK inhibitors can suppress the abnormal activation of immune cells: inhibition of JAK3 reduces effector T cell activation mediated by γc family cytokines; inhibition of JAK2/Tyrosine kinase 2 (TYK2) attenuates macrophage activation and proinflammatory cytokine release; and inhibition of JAK1/JAK3 limits B cell activation and autoantibody production. Together, these actions synergistically mitigate immune cell–mediated tissue injury (21). This provides a mechanistic rationale for the application of JAK inhibitors in autoimmune and inflammatory diseases (22).

A study by Salem et al. (18) showed that early administration of high-dose abatacept combined with the selective JAK inhibitor ruxolitinib in patients with severe ICI associated myocarditis significantly improved the survival rate, reducing the mortality rate from 60% to 3%.The study also analyzed cardiac tissue from ICI associated myocarditis model mice and the RNA sequencing data of clinical patients’ myocardial tissue in another study (23), and found that the expression of genes related to the JAK-STAT signal transduction pathway was upregulated, providing a strong theoretical support for the treatment of ICI associated myocarditis with JAK inhibitors. Tofacitinib, a first-generation small-molecule JAK inhibitor, selectively inhibits the tyrosine kinase activity of JAK1 and JAK3 subtypes (19). By blocking the signals of pro-inflammatory factors (such as interleukins, interferons) and immune-related cytokines (such as γc cytokine, chemokine CXCL10, etc.) that depend on JAK-STAT pathway transduction, it reduces T cell activation, downstream pro-inflammatory gene expression, and immune-related cytokines production (20). Thus, compared with other immunosuppressants, it may therefore be more suitable for patients with concurrent multi-organ irAEs who are intolerant to MMF.

Several clinical studies and case reports support the clinical utility of tofacitinib in steroid-refractory ICI associated myocarditis. A retrospective study from Zhongshan Hospital of Fudan University, enrolled 24 patients with ICI associated myocarditis. Among them, 11 patients who responded poorly to glucocorticoids received tofacitinib (5 mg twice daily) (19). Seven of these patients achieved clinical recovery, with a marked reduction in the key cardiac injury biomarker troponin. Additionally, a case report described a patient with stage IV nasopharyngeal carcinoma receiving a PD-1 inhibitor combined with chemotherapy (24). Despite treatment with high-dose corticosteroids and intravenous immunoglobulin, the patient’s myocardial biomarkers remained persistently elevated. However, after one month of combination therapy with tofacitinib (5 mg twice daily), the levels of hs-TnT, CK-MB, and inflammatory factors (IL-6, IFN-γ) all approached normal, without the occurrence of severe cardiac adverse events. Previous cases and studies on the use of tofacitinib/JAK inhibitors for the treatment of ICI associated myocarditis, as well as the present case, are summarized in **Table 1**.

**Table 1:** Previous reported cases/studies of tofacitinib/JAK inhibitors treatment for ICI-associated myocarditis, including the present case.

Author	Year	Number of Patients	Initial Treatment Failure	JAK Inhibitor (Dose/Course)	Outcomes	Infection Status
Liu et al. (20)	2020	2	Patient 1: high-dose steroids (500 mg × 4 days) + plasma exchange (Day 4, Day 7); persistent dyspnea	Tofacitinib 5 mg BID; duration: 30 days	Cardiac biomarkers improved; discharged; no tumor recurrence	Both not reported
			Patient 2: steroids (1 mg/kg × 3 days) + IVIG (15 g/day × 3 days); hs-TnT rising	Tofacitinib 5 mg QD; duration: 2 months	Clinical symptoms improved; none adverse events	
Wang et al. (19)	2021	11	Steroids (40–500 mg/day) tapering; hs-TnT rising	Tofacitinib 5 mg BID; duration not specified	7 patients recovered after treatment; 2 died from severe pneumonia; 2 died from progressive myocarditis	2 patients developed septic shock due to severe pneumonia
Xing et al. (24)	2022	1	High-dose steroids (500 mg × 3 days) + IVIG (0.4 g/kg × 5 days); hs-TnT rebound	Tofacitinib 5 mg BID; duration: 1 month	Hs-TnT declined; cardiac symptoms improved; tumor recurrence	Not reported
Salem et al. (18)	2023	18	Control group: High-dose steroids (≥500 mg × ≥2 days), continued deterioration; then plasma exchange, low-dose abatacept, or MMF; mortality 60%	Ruxolitinib 15/10/5 mg BID (exact dose not specified) + high-dose abatacept	17 patients recovered; 1 died from respiratory failure (refusing intubation)	Not reported
present case	2025	1	High-dose steroids (500 mg × 3 days) + IVIG (20g × 7 days) + MMF 0.5g BID; hs-TnT rebound	Tofacitinib 5 mg BID; duration: 1 month	Hs-TnT declined; cardiac symptoms improved; no tumor recurrence	fungal infection

IVIG, intravenous immunoglobulin; MMF, mycophenolate mofetil; hs-TnT, high-sensitivity troponin T; BID, bis in die (twice daily); QD, quaque die (once daily).

In this report, we present a complex case of severe, multi-organ irAEs, including steroid-refractory myocarditis, induced by the bispecific antibody QL1706.Despite first-line therapy with high-dose glucocorticoids and intravenous immunoglobulin, the myocardial injury persisted, followed by endocrine involvement (hypothyroidism). Subsequent treatment with glucocorticoids and MMF then led to secondary adrenal insufficiency, hematological suppression, and electrolyte disturbances. Faced with this life-threatening multi-system deterioration, a multidisciplinary team decided to add the JAK inhibitor tofacitinib. This intervention was followed by a rapid and significant decline in cardiac-specific troponin levels, alongside overall clinical improvement, underscoring its potential efficacy in this steroid-refractory setting.

Nevertheless, the safety profile of tofacitinib in cancer patients warrants careful consideration. Firstly, tofacitinib’s broad immune-suppressive effects may compromise the body’s anti-infective capacity, increasing susceptibility to bacteria, viruses, fungi, and other pathogens (25). Since patients receiving ICIs therapy have altered immune function, the addition of tofacitinib may further elevate infection risk, which is the most concerning safety issue. This risk is critically amplified in patients already under profound immunosuppression from high-dose steroids and/or other agents like MMF. In the present case, after tofacitinib administration, the patient developed oral ulcers, diarrhea, and pulmonary fungal infection, which resolved with supportive management and appropriate antimicrobial therapy. Therefore, vigilant monitoring for infection and implementation of appropriate prophylactic strategies are indispensable components of management when employing tofacitinib in patients with cancer and complex irAEs.

Secondly, the potential oncologic impact requires careful evaluation. As long-term oncologic follow-up data are currently unavailable, the effect of short-term intensive immunosuppression on tumor progression cannot yet be determined. Tofacitinib was administered to control life-threatening ICI-induced immune toxicity rather than to directly suppress antitumor immunity. Previous studies suggest that temporary interrupting antitumor therapy may carry a relatively low short-term risk of progression (26). Moreover, deficient mismatch repair (dMMR) tumors may retain partial antitumor immune surveillance after brief immunosuppression (27). Studies in autoimmune diseases suggest that long-term use of tofacitinib (with a median follow-up of 4 years) may increase the risk of malignant tumor development (28). However, this consideration is particularly crucial for patients with active malignancies. Furthermore, tofacitinib may induce other adverse effects such as gastrointestinal discomfort and hematological abnormalities (29).

In summary, the observed symptomatic improvement and marked decline in cardiac biomarkers in this case support the potential efficacy of tofacitinib in steroid-refractory ICI associated myocarditis. However, given the insufficient evidence and absence of prospective standardized protocols, tofacitinib should currently be used as a rescue therapy for cases unresponsive to first-line glucocorticoids. Further high-quality clinical studies are warranted to better define its role, optimal dose, and treatment duration in the management of severe irAEs.

## Data Availability

The raw data supporting the conclusions of this article will be made available by the authors, without undue reservation.
